# Study on correlation between Chinese medicine syndromes in stroke and neurological deficits during recovery phase: Perspective

**DOI:** 10.1097/MD.0000000000039600

**Published:** 2024-09-27

**Authors:** Hao Ren, Ai-hua Yang, Yi-sheng Cai, Yi Qin, Tong-you Luo

**Affiliations:** a Department of Rehabilitation, Fengdu People’s Hospital, Chongqing, China; b Department of Gastroenterology, Fengdu People’s Hospital, Chongqing, China.

**Keywords:** neurological deficit, stroke, syndrome, traditional Chinese medicine

## Abstract

Stroke is a leading cause of long-term disability and mortality worldwide, necessitating effective rehabilitation strategies for successful recovery. Traditional Chinese medicine (TCM) has gained recognition as a complementary and alternative approach in stroke rehabilitation, owing to its unique syndromes that offer valuable insights for personalized treatment plans. This study aims to elucidate the correlation between TCM syndromes observed during the recovery phase of stroke and the associated neurological deficits. Syndromes such *as Blood stasis, Phlegm-dampness, Qi deficiency, and Yin deficiency* were systematically examined, while standardized neurological assessments, encompassing motor function, sensory perception, and cognitive abilities, were employed to evaluate the extent of neurological impairment. Rigorous statistical analyses were conducted to discern potential correlations between TCM syndromes and the severity of neurological deficits. The results revealed statistically significant positive associations between certain TCM syndromes, particularly *Blood stasis* and *Phlegm-dampness*, and heightened neurological deficits during the recovery phase post-stroke. These findings suggest that these syndromes may serve as indicators of more severe brain injury post-stroke, thereby guiding the development of tailored rehabilitation strategies. By establishing robust connections between TCM syndromes and neurological deficits, this study contributes to advancing our understanding of stroke recovery through an integrated approach that incorporates TCM principles. Moreover, it underscores the potential benefits of integrating TCM into conventional rehabilitation protocols, offering valuable insights for healthcare professionals and potentially improving patient outcomes.

## 1. Introduction

Stroke, a major cause of significant disability and mortality worldwide, manifests through acute symptoms such as fainting, hemiplegia, facial asymmetry, slurred speech or aphasia, and numbness on one side of the body.^[1]^ Primarily affecting middle-aged and elderly individuals, it rapidly deteriorates the quality of life and imposes substantial economic and societal costs.^[2,3]^ As reported by the China Stroke Association, the economic impact of stroke in China was estimated at approximately 40 billion *Yuan* in 2015.^[4]^ Moreover, the China National Cerebrovascular Disease Screening Survey highlighted an incidence rate of 329 strokes per 100,000 individuals aged 40 to 74 between 2007 and 2013.^[5]^ The *2018 China Health and Health Statistics Yearbook* noted that strokes accounted for more than 20% of all deaths related to cerebrovascular diseases in 2017, indicating that stroke is responsible for roughly one in every 5 deaths in the country.^[6]^

Despite extensive prevalence, strokes have a low cure rate and are associated with a prolonged disease course, resulting in significant long-term disabilities for over 3-quarters of survivors.^[7]^ The Global Burden of Disease study from 2019 underscores an increase in both the incidence and mortality rates of stroke globally from 1990 to 2019, despite reductions in age-standardized rates, notably among individuals older than 70.^[8]^ This research particularly highlights the disproportionate burden of stroke in low-income countries and points out high body mass index as a rapidly emerging risk factor, emphasizing the critical need for effective primary prevention measures to mitigate the global stroke burden.^[8]^

Further detailed analysis conducted by Tu WJ et al revealed that in 2020, the prevalence of stroke in China was 2.6%, with an incidence rate of 505.2 per 100,000 person-years and a mortality rate of 343.4 per 100,000 person-years.^[9]^ These statistics not only underscore the profound impact of stroke on China’s public health but also stress the urgent need for more effective preventive strategies. Additional studies since 2015 indicate concerning trends in hypertension and diabetes as significant risk factors, reinforcing the importance of robust stroke prevention and management initiatives. This ongoing concern led to the initiation of the Healthy China 2030 Stroke Action Plan, a critical step toward addressing this pressing public health issue.^[10]^

Traditional Chinese Medicine (TCM) attributes the onset of stroke to disruptions in the balance between *Yin* and *Yang* and the flow of *Qi* and *Blood* within the body. TCM theory posits that pathogenic factors, such as *Wind*, *Heat*, *Phlegm*, and *Blood* stasis, block the meridians, thereby impairing cerebral function and precipitating a stroke.^[11]^ The pathogenesis of stroke in TCM is multifaceted, often involving a coexistence of deficiency (e.g., *Qi, Blood, or Yin* deficiency) and excess patterns (e.g., *Phlegm* accumulation or *Blood* stasis).^[11]^ In 2012, the Medical Administration Department of the National Administration of Traditional Chinese Medicine issued the “Clinical Pathway for 22 Specialties and 95 Diseases,” which further standardized the diagnosis and treatment of stroke.^[12]^ This helped to improve the diagnostic criteria for TCM patterns in stroke patients.

Previous studies underscore the prognostic significance of TCM syndromes in the context of ischemic stroke recovery during the acute phase.^[13–16]^ Research indicates that the presence of *Fire-heat*, *Qi* deficiency, and *Blood* stasis syndromes are closely associated with increased severity of neurological deficits and impaired recovery outcomes.^[13,14]^ These findings suggest that such syndromes can serve as critical indicators for the severity of stroke and potential complications, guiding more targeted therapeutic strategies. Complementarily, it has been revealed that *Phlegm-heat* syndrome consistently correlates with higher levels of neurological impairment over time, highlighting its enduring impact on patient health.^[15]^ Furthermore, interactions between specific syndromes, such as *Qi* deficiency, and coagulation functions have been investigated, suggesting that these syndromes may influence stroke pathology through effects on coagulation pathways.^[16]^ Together, these studies advocate for the integration of TCM syndrome differentiation into standard stroke treatment protocols, promoting a nuanced approach that enhances predictive accuracy and optimizes patient-specific therapeutic outcomes.

Current research primarily focuses on the correlation between TCM syndromes and neurological deficits during the acute phase of stroke. However, there is a discernible lack of comprehensive studies examining these relationships specifically during the stroke recovery phase. This research gap is critical, as it suggests a potential area for further investigation. Consequently, the principal contribution of this study is its detailed exploration of the specific correlations between TCM syndromes and neurological deficits throughout the recovery phase of stroke. Our analysis reveals a significant positive correlation between particular TCM syndromes, notably *Blood stasis* and *Phlegm-dampness*, and the severity of neurological impairments. These findings could significantly influence the development of tailored rehabilitation programs, offering a foundation for enhancing therapeutic strategies based on individual syndromic presentations, thereby potentially improving recovery outcomes for stroke survivors.

## 2. Evolution pattern of stroke syndrome

The progression of stroke syndromes in TCM reflects a dynamic interplay between the body’s responses and pathogenic factors over time. Initially, acute stages may be characterized by *Wind* and *Heat* syndromes, which obstruct *Qi* and *Blood* flow. As the condition advances, *Blood* stasis and *Phlegm* syndromes become more prominent, particularly during the recovery phase. Understanding these evolving patterns is critical for implementing timely and syndrome-specific interventions that restore balance and facilitate recovery. According to the statistical analysis conducted by Yang L et al,^[17]^ among 1418 stroke patients, the recovery period of stroke is mainly characterized by *Blood stasis* syndrome and *Phlegm* syndrome, with a higher frequency of *Qi* deficiency syndrome compared to the *Heat* syndrome. In the post-stroke sequelae period, *Blood* stasis syndrome, *Qi* deficiency syndrome, and *Phlegm* syndrome are the main syndromes observed. Both *Blood* stasis syndrome and *Phlegm* syndrome have a significantly high proportion in each period. Wang SD et al^[18]^ found that 2 weeks after the occurrence of stroke, *Blood* stasis syndrome is more common, while *Qi* deficiency syndrome is frequently observed. In the study conducted by Zhou WQ et al^[19]^ involving 127 stroke cases, the distribution of syndrome patterns over time indicated that within 14 days of onset, *Wind* syndrome, *Heat* syndrome, and *Phlegm* syndrome are commonly seen, especially *Wind* syndrome. After 30 days, *Qi* deficiency and *Blood* stasis syndromes become more prominent. It can be observed that *Wind* syndrome is the most important and common syndrome in the initial stage of stroke. The probability of *Wind* syndrome occurrence gradually decreases after the onset of the disease. *Phlegm* and *Blood* stasis are the 2 main pathological factors in stroke, often coexisting. The combination of *Phlegm* and *Blood* stasis is the fundamental pathogenesis of stroke and is present throughout the disease.

## 3. Research on factors influencing stroke syndromes

A study conducted by Yu XQ et al^[20]^ analyzed the clinical data of 1340 patients and discovered that several factors have an impact on the occurrence and changes in stroke syndromes. Unhealthy lifestyle habits are risk factors for the occurrence of the “*Heat* syndrome,” while triglycerides and retirement act as protective factors for the “*Heat* syndrome.” Smoking and body mass index are risk factors for the “*Phlegm*-dampness syndrome.” Patients who are workers, have a family history of stroke, and have high fasting blood sugar levels have a lower frequency of developing the “*Blood* stasis syndrome.” On the other hand, patients with longer disease duration and hyperlipidemia have a higher frequency of developing the “*Blood* stasis syndrome.” Patients who are workers, have a family history of stroke, and are overweight have a lower probability of developing the “*Qi* deficiency syndrome,” while males and long-term stroke survivors are more prone to develop the “*Qi* deficiency syndrome.” Hypertension, hyperlipidemia, excessive salt intake, being underweight, or having high fasting blood sugar levels increase the likelihood of developing the “*Yin* deficiency with *Yang* hyperactivity syndrome,” while body mass index acts as a protective factor. In addition, ischemic stroke patients are more likely to present with *Wind* syndromes, *Blood* stasis syndromes, and *Qi* deficiency syndromes, while hemorrhagic stroke patients have a higher frequency of the “*Heat* syndrome.” Another study by Liang WX et al^[21]^ found that male patients had significantly higher mean values and proportions of *Phlegm* syndromes compared to female patients. The proportion of *Blood* stasis syndromes in the 60 to 69 age group was significantly higher than in other age groups. Physical laborers had significantly higher mean values and proportions of *Wind* syndromes compared to other types of labor, while intellectually laborers had significantly higher mean values and proportions of wind syndromes compared to other types of labor.

## 4. Stroke syndromes and neurological dysfunction

Recent studies have increasingly focused on the relationship between TCM syndromes and neurological dysfunctions in stroke patients. Specific syndromes, such as *Qi* deficiency, *Blood* stasis, and *Phlegm*-dampness, are associated with distinct patterns of neurological impairment. For instance, *Qi* deficiency often manifests as significant fatigue and general weakness, while *Blood* stasis is linked to exacerbated motor dysfunction due to impaired circulation. Recognizing these correlations is essential for developing targeted TCM interventions that address the specific neurological deficits associated with each syndrome. Based on the distribution of TCM syndrome and combined with the National Institutes of Health Stroke Scale (NIHSS) and the Barthel Index (BI) scores,^[22]^ the study explored the relationship between TCM syndrome elements and neurological functional impairment as well as daily activities in the recovery phase of stroke patients. The findings of this study will provide scientific and objective evidence for TCM rehabilitation treatment during the recovery phase of stroke and the prognosis of stroke patients.

According to Zhang Y’ study, the timing of the occurrence of *Qi* deficiency syndrome in ischemic stroke is closely related to the degree of neurological functional impairment in patients.^[23]^ Xin XY et al conducted a comprehensive analysis of clinical information, including stroke syndrome diagnostic scales, National Institutes of Health Stroke Scale scores, and Barthel Index scores collected at 5 different time points during the course of stroke in 706 stroke patients.^[24]^ Their retrospective study combined with relevant regression analysis showed that patients with qi deficiency syndrome in stroke had more severe neurological functional impairment than those without *Qi* deficiency syndrome.^[24]^ Comparing the different syndrome elements in the acute phase of stroke, it was found that *Qi* deficiency syndrome was closely related to poor long-term prognosis.^[24]^ Therefore, early intervention is recommended. Lin JX’s study has reported that if patients with ischemic stroke in the acute phase develop a heat syndrome within 3 days of onset, they are more likely to experience complications during hospitalization.^[25]^ The degree of neurological functional impairment and evaluation of daily living function status will also be more severe in patients who develop heat syndrome earlier or do not develop it at all, and their recovery ability upon discharge will be worse.^[25]^

## 5. Stroke syndrome and prognosis

The differentiation of syndromes in traditional Chinese medicine includes the description of the development stage and severity of the disease, and therefore can be considered as a description of the prognosis for the patient. According to Zhou WQ et al,^[26]^ the severity of the syndrome directly reflects the severity of the condition. Their research suggests that the prognosis is relatively worse for patients with the syndromes of *Wind*, *Phlegm*, excessive *Heat*, and *Qi* deficiency, compared to those with the syndromes of *Blood* stasis and *Yin* deficiency with excessive *Yang*. The more complex the syndrome, the worse the prognosis. *Qi* deficiency with *Yang* collapse or *Yin* deficiency with excessive *Yang*, accompanied by *Wind* and *Phlegm* disturbance, are considered clinically severe syndromes. It is difficult to reflect the severity of the condition and prognosis solely based on differentiation of deficiency and excess. Zhou B.^[27]^ suggested in his study that the improvement rate of the meridian group was significantly higher than that of the *ZangFu* group. The improvement rate of the *Qi-Yin* deficiency and liver *Yang* excessive group was higher than that of the *Phlegm*-dampness and *Blood* stasis group. The mortality rate of the *Phlegm-heat* closed internally affecting the heart group was higher than that of the wind-fire disturbance clearing the mind group.

## 6. Study correlation between stroke syndrome and objective indicators

The correlation between the syndrome of stroke and objective indicators. The diagnosis of syndrome is based on the external manifestations of the disease. The advancement of diagnostic methods has led to research on the correlation between TCM syndromes and various objective indicators. Some scholars have conducted experiments and found connections between syndromes and certain indicators, attempting to find specific indicators that are characteristic of syndromes, or even uncover the material essence of syndromes. However, there are also scholars who have obtained negative results and believe that Chinese and Western medicine are 2 completely different theoretical systems with different theoretical foundations and methods. It is seen as far-fetched and forced to relate a certain objective indicator to a specific syndrome.

The phenomenon of TCM patterns is a comprehensive manifestation of the etiology, pathology, location, and development of a disease at a certain stage. The mechanism behind the formation of TCM patterns is exceptionally complex, influenced by countless factors. This complexity poses significant challenges in studying TCM patterns. As a scientific study, rigorous design and strict control of interfering factors are needed, which is extremely difficult to achieve in clinical practice. Furthermore, traditionally, TCM pattern diagnosis relies on the sensory perception and judgment of the physician, rather than relying on instrumental examination. This means that the description of TCM patterns in TCM theory is highly condensed and generalized. Nevertheless, we believe that even in the presence of numerous interfering factors, with the use of modern scientific research methods and statistical analysis, we can still discover some simple regularities. This is also the prerequisite and foundation for understanding the complex principles underlying TCM patterns.

## 7. Clinical evidence of TCM in treating stroke

### 7.1. Single herb medicine

Single herb treatments in TCM have demonstrated promising results in stroke therapy. Clinical trials have shown that the aqueous extract of saffron (*Crocus sativus* L.) effectively modulates oxidative stress in ischemic stroke patients, indicative of its potential neuroprotective effects.^[28,29]^ Furthermore, Ginkgo biloba extract has been validated to enhance cognitive and neurological functions in patients with ischemic stroke, supporting its utility in aiding brain function recovery.^[30,31]^ Pomegranate supplementation has also been documented to facilitate cognitive and functional recovery post-stroke.^[32]^ Additionally, extracts from Radix/rhizoma notoginseng (sanchitongtshu) have been reported to exhibit positive effects in the treatment of ischemic stroke, underscoring the potential of single herb medicines in stroke rehabilitation.^[33]^

### 7.2. Chinese herbal formulas

Chinese herbal formulas are carefully crafted combinations of herbs that work synergistically to address the multifaceted nature of stroke. These formulas are designed to harmonize the body’s internal environment by simultaneously targeting various pathological factors. For example, the Qizhitongluo capsule is formulated to promote blood circulation and resolve *Phlegm*, thereby addressing both *Blood* stasis and *Phlegm*-dampness syndromes.^[34]^ This integrative approach reflects TCM’s strength in providing comprehensive treatment strategies that align with the principles of syndrome differentiation and holistic care. Gegen Tongluo capsules not only alleviate symptoms in the recovery phase of ischemic stroke but also effectively manage primary hypertension.^[35]^ Additionally, formulations such as Tiaoqiheying decoction and Sanhuang Xiexin decoction have been shown to significantly enhance functional recovery in acute ischemic stroke.^[36,37]^ The integration of Dengzhan Shengmai capsules has positively impacted the secondary prevention of ischemic stroke,^[38]^ and the beneficial effects of Di-huang-yin-zi on stroke patients have been validated through randomized controlled trials.^[39]^

### 7.3. Current research on Chinese medicines

Ongoing research is exploring the therapeutic potential of various Chinese medicines and formulations in stroke management. Danzhi Xiaoyao Powder is currently under study for its efficacy in treating post-stroke depression, highlighting the potential for Chinese medicine in mental health applications.^[40]^ Peony and licorice decoction fumigation treatments are being investigated for functional disorders such as strephenopodia following stroke.^[41]^ Buyang Huanwu Tang has shown potential in addressing post-stroke fatigue, suggesting significant benefits in enhancing patients’ quality of life.^[42]^ Additionally, clinical trials have confirmed the positive effects of Xueshuan Xinmai tablets on brain functional connectivity in acute ischemic stroke, further validating the role of TCM in comprehensive stroke care.^[43]^

### 7.4. Theoretical basis for TCM in enhancing stroke prognosis

The application of TCM in stroke recovery is increasingly supported by scientific research, which elucidates the diverse biological mechanisms through which TCM affects treatment outcomes. Notably, TCM employs complex, multi-target strategies that extend beyond symptomatic relief to modulate underlying molecular and cellular pathways.

Studies on the aqueous extract of saffron (*C sativus* L.) have demonstrated its effectiveness in reducing oxidative stress in ischemic stroke patients, which supports its potential neuroprotective properties.^[28,29]^ Similarly, Ginkgo biloba extract has been documented to improve cognitive and neurological functions, highlighting its neuroprotective capabilities.^[30,31]^

Investigations into pomegranate supplementation and notoginseng-ginseng extracts (sanchitongtshu) also illustrate their capacity to promote recovery after stroke. These effects are likely mediated through their influence on anti-inflammatory and antioxidant pathways, suggesting that TCM can facilitate neurological recovery by regulating key biochemical pathways.^[32,33]^

Moreover, Chinese herbal formulas such as Qizhitongluo and Gegen Tongluo capsules showcase the advantage of using multi-herb combinations. These formulas have not only improved symptoms during the recovery phase of ischemic stroke but have also shown efficacy in managing primary hypertension, illustrating the holistic approach of TCM in treating complex health conditions.^[34,35]^

In addition, research into Buyang Huanwu Decoction combined with RT-PA intravenous thrombolysis for Qi deficiency and blood stasis types of stroke exemplifies TCM’s capability to enhance functional recovery. This particular treatment modality has been shown to activate the Keap1-Nrf2/ARE antioxidant pathway, thereby providing significant neuroprotection and enhancing the neuronal survival environment, which contributes to improved recovery processes.^[44]^ This indicates TCM’s potential role in mitigating neurological damage by modulating oxidative stress responses within the body.

### 7.5. Efficacy and mechanism of An-Gong-Niu-Huang Wan’s in stroke treatment

An-Gong-Niu-Huang Wan (AGNHW) is increasingly recognized for its neuroprotective capabilities, particularly in the context of stroke treatment. Rigorous clinical and experimental research has substantiated its efficacy, showcasing significant therapeutic benefits across diverse studies.

### 7.6. Clinical efficacy in hemorrhagic stroke management

In a methodologically robust study by Feng Huiyuan at Hunan University of Chinese Medicine, AGNHW was evaluated for its effectiveness in managing intracranial hypertension in patients with hemorrhagic stroke due to phlegm-heat internal obstruction syndrome. The randomized clinical trial involved 40 patients divided into a treatment group, which received AGNHW alongside standard care, and a control group. Results indicated substantial improvements in intracranial pressure, neurological function, reduction in brain edema, and decreases in inflammatory markers TNF-α and IL-6. Notably, the treatment group achieved a 95% effective rate in symptom improvement, significantly higher than the 75% in the control group, demonstrating the clinical utility of AGNHW in acute hemorrhagic stroke.^[45]^

### 7.7. Protective mechanisms against ischemic damage

Zhang S. et al explored the pretreatment effects of AGNHW on cerebral ischemia in rats in a study published in Front Pharmacology. The research, focusing on the GSK-3β/HO-1 pathway, revealed that AGNHW pretreatment significantly reduced neurological deficits and infarct size, and enhanced the brain’s antioxidant capacity. These findings suggest that the protective effects of AGNHW are mediated through its antioxidant properties, particularly through the activation of the GSK-3β/HO-1 pathway, offering a promising strategy for ischemia prevention.^[46]^

### 7.8. Impact on apoptotic pathways

In further research featured in the Journal of Ethnopharmacology, Wang GH et al demonstrated that AGNHW modulates apoptosis-related proteins following cerebral ischemia. The formula significantly reduced the levels of pro-apoptotic proteins Bax and caspase-3 while increasing the anti-apoptotic protein Bcl-2. This modulation of apoptotic pathways suggests that AGNHW effectively prevents neuronal cell death, contributing to its neuroprotective effects in clinical settings.^[47]^

These studies collectively affirm AGNHW’s effectiveness in reducing neurological impairments through anti-inflammatory, anti-apoptotic, and antioxidant mechanisms. This evidence not only supports the traditional use of AGNHW but also underscores its potential for integration into contemporary stroke treatment protocols. Further clinical trials are warranted to optimize its application and fully understand its therapeutic mechanisms in modern medical practice, enhancing its integration into integrated treatment protocols for stroke recovery.

Overall, the therapeutic efficacy of TCM in the context of stroke treatment and recovery is underscored by its ability to interact with multiple signaling pathways, thereby offering a comprehensive approach to enhancing patient outcomes. These findings not only validate the role of TCM in a clinical setting but also pave the way for its integration into conventional treatment paradigms, providing robust evidence for its continued research and application in modern healthcare settings.

## 8. Summary

This study reveals significant correlations between TCM syndromes, particularly *Blood* stasis and *Phlegm-dampness*, and the severity of neurological deficits during the stroke recovery phase. These findings have important implications for clinical stroke rehabilitation.

### 8.1. Application to clinical practice

The correlations identified between specific TCM syndromes and neurological impairments offer practical insights for developing tailored rehabilitation strategies. For example, patients diagnosed with *Blood* stasis syndrome may benefit from interventions specifically designed to enhance blood circulation and resolve stasis, potentially mitigating the severity of their neurological deficits.^[11]^ Similarly, patients exhibiting *Phlegm-dampness* syndrome might respond better to treatments aimed at resolving *phlegm* and reducing *dampness*, which could accelerate their recovery by addressing the underlying pathological mechanisms revealed in this study.^[11]^ These syndrome-specific strategies could lead to more precise and effective rehabilitation outcomes.

### 8.2. Integration with conventional medicine

Integrating TCM principles with conventional stroke rehabilitation practices presents an opportunity to enhance the personalization of treatment plans.^[48]^ By combining the holistic approach of TCM with evidence-based conventional therapies, clinicians can develop comprehensive strategies that address both the physiological and energetic aspects of stroke recovery.^[48]^ This interdisciplinary approach not only bridges traditional and modern medicine but also has the potential to optimize patient outcomes through synergistic effects. However, the practical application of TCM syndromes in a clinical setting requires standardized diagnostic criteria and careful consideration of individual patient responses, as variability in syndrome interpretation and treatment efficacy may pose challenges to widespread implementation.

### 8.3. Potential impact and limitations

While the study’s findings are promising, several limitations must be acknowledged. The variability in how TCM syndromes are interpreted across different practitioners and regions may limit the consistency and reliability of applying these findings in diverse clinical settings. Additionally, the effectiveness of TCM-based rehabilitation strategies can vary among patients, making it essential to approach these interventions with caution. Future research should focus on standardizing TCM syndrome differentiation to ensure consistency in diagnosis and treatment. Furthermore, large-scale, multicenter studies are needed to validate the effectiveness of TCM-informed personalized rehabilitation strategies across varied populations, thereby ensuring their broader applicability and impact in clinical practice.

## Acknowledgments

This study was supported by Chongqing Science and Technology Joint Youth Project (2022QNXM078).

## Author contributions

**Conceptualization:** Hao Ren, Tong-you Luo.

**Data curation:** Hao Ren, Tong-you Luo.

**Funding acquisition:** Hao Ren.

**Investigation:** Tong-you Luo.

**Methodology:** Hao Ren, Yi Qin.

**Project administration:** Tong-you Luo.

**Resources:** Hao Ren, Ai-hua Yang, Yi-sheng Cai, Yi Qin, Tong-you Luo.

**Supervision:** Tong-you Luo.

**Validation:** Hao Ren, Ai-hua Yang, Yi-sheng Cai, Yi Qin, Tong-you Luo.

**Visualization:** Hao Ren, Ai-hua Yang, Yi-sheng Cai, Yi Qin, Tong-you Luo.

**Writing – original draft:** Hao Ren, Ai-hua Yang, Yi-sheng Cai, Yi Qin, Tong-you Luo.

**Writing – review & editing:** Hao Ren, Ai-hua Yang, Yi Qin, Tong-you Luo.
